# Quantifying Bot Impact: An Information-Theoretic Analysis of Complexity and Uncertainty in Online Political Communication Dynamics

**DOI:** 10.3390/e27060573

**Published:** 2025-05-28

**Authors:** Beril Bulat, Martin Hilbert

**Affiliations:** Department of Communication, University of California, 1 Shields Avenue, Davis, CA 95616, USA; hilbert@ucdavis.edu

**Keywords:** dynamical systems, information theory, political bots, social bots, twitter, social media

## Abstract

Bots have become increasingly prevalent in the digital sphere and have taken up a proactive role in shaping democratic processes. While previous studies have focused on their influence at the individual level, their potential macro-level impact on communication dynamics remains underexplored. This study adopts an information-theoretic approach from dynamical systems theory to examine the role of political bots shaping the dynamics of an online political discussion on Twitter. We quantify the components of this dynamic process in terms of its complexity, predictability, and its entropy rate, or the remaining uncertainty. Findings suggest that bot activity is associated with increased complexity and, simultaneously, with more uncertainty in the structural dynamics of online political communication. While our dataset features earlier-generation bots, findings foreshadow the possibility for even more complex and uncertain online politics in the age of sophisticated and autonomous generative AI agents. Our presented framework showcases how this can be studied with the use of information-theoretic measures from dynamical systems theory.

## 1. Introduction

The rise of bots in the digital age has transformed the online ecosystem in profound ways. Originally appearing in Internet Relay Chat networks in the early 1990s [1], bots have evolved into a diverse range of applications since then. From information-gathering bots that constantly scour the web [2] to algorithmic trading bots that engage in financial transactions [3], bots have become an integral part of the digital landscape. Among these, political bots have attracted particular attention due to their potential impact on public opinion and democratic processes [4]. 

Political bots are automated agents specifically tasked with public opinion manipulation. They are the epitome of the larger collection of so-called persuasive tech [5,6]. A multitude of studies so far have examined malicious bot activities and provided evidence of bot propaganda during elections and campaigns around the world [4,7,8,9,10]. Previous work suggests bots are capable of passing as humans and engaging in complex interactions with other users [11,12,13]. Among their manipulative strategies are spreading disinformation and amplifying divisive, polarizing content [14,15,16,17,18]. Although there is abundant evidence of bot engagement on Twitter; however, debates concerning the impact of their malicious activities remain to be settled [10,19,20].

While the majority of previous work focuses on their impact at the individual level, important questions remain at the macro level. For instance, how do bots influence the overall communication dynamics? Bots are based on predictive algorithms that follow (machine learning) rules that algorithmically transform input to output, but does this make online political communication less uncertain (given that bots employ predictive algorithms), or more uncertain? Do bots simplify the arising dynamic or does the communication landscape get more complex with bots? These questions are becoming increasingly important as the recent revolution in generative AI is unlikely to decrease the role of bots [21,22]. On the contrary, it is expected that the new technological possibilities of generative AI will lead to rather complex collective dynamics.

To contribute to a better understanding of these arising dynamics, in this work, we follow a long tradition that views communication as a dynamical process [23,24,25,26] and use the tools rooted in information theory, i.e., Claude Shannon’s “mathematical theory of communication” [27,28], to answer these questions. The merger of information theory with theoretical computer science, which, in its roots, goes back to Kolmogorov [29,30] and Sinai [31], allows us to analyze dynamical systems by quantifying how much information and uncertainty is created and passed along a given process [32]. We adopt this methodological framework to analyze a case of online political discussion and quantify the arising dynamic of the system as a whole in terms of predictability, complexity, and remaining uncertainty. We then assess the relationship between political bot involvement and changes in the components of the process, the complexity, and uncertainty levels of the communicative dynamics.

In line with related findings on editor bots in Wikipedia [33] and trading bots in the stock market [3], our findings reveal that political bots significantly impact the dynamics of online political communication, contributing both to its complexity and uncertainty. The presence of political bots is associated with a less predictable and stable online discourse, which can negatively affect public opinion formation around controversial topics. From a methodological perspective, applying a complex systems lens on political bots and online political communication highlights the need for further research into macro-level bot influence and the importance of considering communication processes as complex dynamic systems. 

## 2. Literature Review

### 2.1. A Brief History of Bots

Bots are automated agents that perform tasks online [34,35]. They can run continuously without requiring any human intervention, though their agency is limited, and they can only act within the boundaries pre-defined in their scripts [36]. While they have surged in popularity and visibility in recent years, their existence goes back to the advent of computing technology [12,35], and they have come a long way since the introduction of Weizenbaum’s Eliza [37], the first chatbot. Today, bots are integral to a wide range of online applications, such as providing virtual customer service or automating content moderation [38,39,40]. Their evolution reflects a broader transformation in their use and overall impact, which greatly expanded with the rise of social media. 

The emergence of social media platforms introduced social bots that automate content production and engagement, blurring the lines between genuine human communication and artificial interaction. Social bots can be defined as automated agents that imitate human behavior on social media [41]. They can serve benign purposes, like news bots that distribute news articles [38,42], or editor bots that oversee published content [36]. Or they can have more insidious purposes, such as spam attacks [43], identity theft [44], or public opinion manipulation [8,45].

A subgroup of social bots, political bots have garnered significant attention for their impact on online discourse [4]. These politically motivated agents have demonstrated their capacity to amplify specific narratives, contributing to the public opinion polarization and posing challenges to the integrity of public discourse [4,8,15]. While several countries have attempted to enact regulations to curb computational propaganda efforts, these attempts largely fell short due to challenges with bot identification and overreliance on platform regulation [46,47]. Meanwhile, the underlying technology continues to evolve rapidly, outpacing regulatory efforts [10,12,48], emphasizing the critical importance of ongoing research in this domain.

### 2.2. Social Bots with a Political Agenda

Although most social bots are automated to carry out simple, repetitive tasks [12], political bots are used for malicious purposes [49,50]. They can monitor user traffic while following a circadian rhythm to mimic real users [11]. The more they act human-like, the more likely they are to receive engagement from humans [13]. They can act alone or coordinated as botnets, amplifying or diminishing targeted viewpoints [48,51]. While their interference in political campaigns and electoral processes has been evidenced around the world [7,8,10,51,52,53,54,55,56,57], they were also found to be actively polarizing across a range of social issues, such as immigration [58], climate change [59], the vaccine debates [14,60], the recent COVID-19 outbreak [61,62,63], and the war in Ukraine [64]. 

A large part of the previous work on algorithmic manipulation has focused efforts on bot detection methods, which have evolved significantly since the initial attempts in 2010 [45,65,66]. Over time, researchers have explored different detection strategies, grouped under three main categories: the crowd-based approach, the network-based approach, and the feature-based approach [12,67]. Crowdsourcing approaches rely on human judgment to identify bots, but this approach comes with potential human annotation biases, in addition to issues with scalability and user privacy [12,68]. Network-based detection involves analyzing the structure of social networks and detecting automated agents through their connection patterns [69]. This approach assumes bots establish close connections with each other, but the literature suggests this approach may fail to capture automated agents integrated within genuine online communities [8]. Feature-based detection systems leverage a wide array of account characteristics to assess automation through supervised machine learning [70,71]; however, this approach necessitates continuous refinements to remain effective due to the ever-evolving nature of bots [72,73]. Recent work comparing bot detection strategies revealed that different approaches can produce markedly different results, further compounding the issue, making cross-study comparisons difficult [74,75]. Overall, bot detection remains an ongoing challenge, requiring constant adaptation from the research community. 

Another major theme in the previous literature was bot influence over discussion networks. Bots were found to actively disseminate misinformation during elections [8,45], amplifying the reach of low-credibility content such as fake news [48]. Studies show that bots spread polarizing content, fostering fear, hate, and violence [51,76]. They are centrally positioned in the discussion networks to influence ongoing discourse through retweets, especially during divisive events [8,48,77,78]. Even weakly connected bots can impact discussions by engaging with influential users [51], alter network sentiments [79], sway network dynamics via their followers [80], and indirectly shape discussions by influencing search engines and recommender systems [81]. 

However, it should be noted that the effectiveness of bot manipulation is contested. Bastos and Mercea [7] suggested that their influence over the entire discussion may be overstated, while a more recent study showed that bots occupy less central positions within the discussion networks, compared to verified influential accounts [20]. There is evidence to suggest that, while bots do amplify certain divisive narratives, they fail at diffusion, as most of their content fails to reach new users beyond their existing audience [52]. Users that are already predisposed to extreme views are most likely to encounter bot-generated content, suggesting minimal shifts in overall public opinion and sentiment [82]. 

At the individual level, research demonstrates that individuals exhibit perceptual biases concerning the prevalence and impact of bots, exaggerating their presence and others’ susceptibility to their influence [83]. Partisan-motivated reasoning also seems to play an important role, as users are more likely to engage with human-like bot accounts when they share the same partisanship [13] and less likely to examine the account when they share the same stance [84]. Despite minimal direct interactions between bots and users on Twitter, a study of over 4000 users highlighted bots’ substantial influence on opinions through indirect exposure, especially on contentious topics [85]. This underscores a general lack of proficiency among users in distinguishing bots from humans [74], often leading to misidentification [86].

Although there is ample evidence regarding bot activity on Twitter, debates about the impact of these malicious activities are yet to be settled [19,20,80]. While some researchers have been warning about their increasing sophistication [41], and recent evidence suggests that there is the potential for political bots to get more advanced [65,87], others have found that the majority of the currently available commercial services and tools only provide rather simplistic and repetitive automation [88]. Most of the Twitter bots in a recent study were found to be “spammers”, with no advanced capabilities and limited intelligence [88]. However, this does not necessarily indicate that their limited capacities could cause no harm. 

One possible impact of political bots could be on the overall communication process as a whole, a potential that has largely been underexplored in previous work. 

### 2.3. Online Political Communication as a Dynamical Process

Political bots, like all other bots, are automated scripts that follow a predefined path and act within a defined rule set. In a way, they epitomize the concept of predictability by design. In addition, at first glance, the deterministic nature of political bots may suggest a potential to diminish uncertainty in online political communication, making it more predictable through repetitive actions governed by the parameters preset by their developers. Bot activity, by its preprogrammed, repetitive essence, should logically reduce the complexity of online interactions. However, despite their inherently predictable behaviors, on the macro-level, the presence of political bots in social media may also be introducing a level of complexity and unpredictability into the online political discourse, which could be understood as an emergent phenomenon.

To study this, we use Claude Shannon’s foundational framework [28] of information theory, which introduces the principle that the value of information within a message is inversely proportional to its predictability. In other words, the value of information is quantified not directly by the data transmitted, but by the surprise it delivers to the recipient. The more uncertainty gets reduced, the more information gets communicated. Information is the opposite of uncertainty, and uncertainty can be measured with probability theory. To illustrate, suppose that we are transmitting sequences that include only two characters ‘A’ and ‘B’. The message ‘ABABABA…’ is more predictable than a rather random series of ‘ABBBAABB…’. Repetitive and predictable structures convey less information. If it is more difficult to predict the next letter, there is more uncertainty. 

In this sense, information theory has long been applied to quantify predictable patterns in the analysis of dynamical systems. This makes it possible to quantify how much information and uncertainty are created and passed along at each step of a dynamical process. The methods derived have found applications in a variety of fields, such as physics [89], biology [90], neuroscience [91], psychology [92], and communication [3]. In this framework, the unpredictable aspect of a process is quantified with the traditional concept of remaining uncertainty (i.e., the entropy rate), as proposed by Shannon [28]. The amount of predictable structure inherent in the process can be quantified by predictive complexity. Computational mechanics [93] conceptualizes it as the ‘minimum size, maximally predictive machinery’ that can recreate the structural pattern. Its entropy is the predictive complexity, i.e., the complexity required to produce the predictable pattern [89]. 

Although this approach has relevance to various aspects of communication science, only a few scholars have explicitly utilized this framework to analyze the multi-level dynamics of online communication [94]. Consequently, despite the fact that Shannon’s original paper is literally called “A Mathematical Theory of Communication” [28], the application of information theory to communication as a dynamical system has been limited. In this study, we consider online political communication as an example of a dynamic process. 

### 2.4. Research Questions

On one hand, the pre-programmed simplicity of traditional political bot behaviors, and the premise that they are used to elicit predictable outcomes, as evidenced in the previous work, might suggest a decrease in the complexity and uncertainty of communication dynamics. Most bots engage in simple amplification tasks, such as liking and resharing existing content to simulate social contact [73,78,88]. Considering that they can efficiently perform such repetitive tasks in bulk and at a much higher frequency than human users [78,95], it could be argued that political bots are likely to make the overall communication dynamics more predictable, thereby decreasing the complexity and uncertainty inherent in the communicative process. 

Conversely, the evidence regarding their capability to trigger emotional contagion around negative topics [63], or in spreading polarizing, sensational content [48,62,68], suggests a compelling counterargument. Such activities by political bots may be introducing significant levels of complexity and unpredictability into online political communication, seemingly increasing the system entropy. 

Viewed through the lens of information theory, the level of complexity in online political communication can be determined by the frequency of recurring patterns that emerge during the dynamic process [89], while the remaining uncertainty is the measure of average randomness that is not captured by the structured complexity of the process [28]. It has been previously shown that social bots can have an impact on dynamic processes. In studies on the communicative turns of edits on Wikipedia [33] and trading patterns in foreign exchange markets [3], Hilbert and Darmon reported that algorithmic bots made the structural dynamics of the communicative process more predictable, more complex and, at the same time, more uncertain. They found that bots resolved much of the previous uncertainty, but they also fine-grained the dynamic, both in space (variety) and time (patterns), which created new complexity and induced new uncertainties in the process. 

However, editor and trading bots operate in an entirely different context than a Twitter bot with a political agenda, and as a result, their respective impact may vary significantly. In this work, we adopt the same approach and expand on this analytical framework to examine the role of political bot activity in online political discussions. Unlike previous studies, we delve into the underlying complexity and uncertainty these digital agents introduce into the online political discussion. By quantifying the information dynamics of online political interactions, we measure the complexity and uncertainty involved in the process and seek to answer the following questions: 

RQ1: How is the presence of bots associated with the level of complexity in online political communication dynamics?

RQ2: How is the presence of bots associated with the level of remaining uncertainty in online political communication dynamics?

## 3. Data and Methods

In this study, we perform a multiple regression analysis with two core information-theoretic measures: predictive complexity (C) (also known as statistical complexity) [96,97] and remaining uncertainty (h) (in form of the sequences’ entropy rate) [29,30]. They offer complementary insights into the temporal structure of online discourse. In simple terms, predictive complexity captures the amount of historical structure required to predict future behaviors, while remaining uncertainty quantifies how much unpredictability remains even after accounting for previous patterns. Together, they allow us to quantify how structured, and yet how random, a conversation stream gets over time.

To apply these measures, we first transform our tweet data into discrete categorical sequences based on emotional content. These sequences are then analyzed using the Causal State Splitting Reconstruction (CSSR) algorithm [98] in Darmon’s implementation [99], which infers the underlying patterns and randomness within each emotional stream. The resulting C and h values are expressed in bits, which allows us to compare how the presence of bots affects the structural complexity and uncertainty of online political discourse across 650 time-based emotional sequences.

Aside from the two information-theoretical measures as our dependent variables (quantifying how complex and how uncertain the communication dynamics are), we also include bot-level as our explanatory independent variable in our regression model and some potential confounders as controls, like word count, character count, word complexity, and time variance.

The data were collected in real time during the first democratic presidential debate in 2019 by leveraging Twitter’s search API with a keyword approach. The dataset provides roughly 395 K tweets that include the keyword ‘#demdebate’. To represent the resulting stream of tweets quantitatively, we semantically analyze them using IBM Watson Developer Cloud’s natural language understanding service, for a representation of each tweet in terms of the five primary emotions conveyed, mainly anger, fear, sadness, joy and disgust [100]. The IBM Watson API is a natural language processing tool that employs machine learning algorithms to extract semantic information from text, including concepts, emotions, and sentiments. Rather than relying on traditional methods, the application utilizes a Recurrent Neural Network to dynamically capture and classify sentiments. The API has been trained on a diverse set of sources, including tweets, and has been used in numerous research projects [101,102].

Since we worked with traditional information theory, we needed to convert the raw scores into categorical variables (otherwise we would have to work with differential/continuous entropy). We assigned each tweet into one of four categorical bins based on its emotional score from IBM Watson, which ranged from 0 to 1. This left us with five temporal sequences of roughly 395 K tweets, one for each emotion (i.e., anger, fear, sadness, joy, and disgust). We wanted to analyze statistical results, so we needed more than five sequences, but our sequences also needed to be sufficiently long, because our nonlinear information-theoretic measures converge rather slowly. We found a statistically robust sampling solution by creating sequences of 3000 consecutive tweets for each emotion, ending up with 650 temporal sequences in total. Despite sampling motivations, it is methodologically useful that each sequence is equally long, as longer/shorter sequences will increase/decrease the likelihood of more structural diversity and more/less uncertainty. 

### 3.1. Binning of Variables

To ensure statistical robustness and gain a nuanced understanding of the data distribution, we employed three different binning strategies in categorizing the emotional scores of each tweet in each sequence. This was deemed necessary to account for variations in the distribution of emotion scores, as how we bin the data into categorical variables might affect our analysis. The first binning strategy involves categorizing each raw value in the sequence by dividing it into quartiles, which creates four categories of approximately equal sizes, where the first category includes values that are less than the 25th percentile and the last category includes values greater than the 75th percentile. The second binning strategy involves sorting and ranking the values in each sequence and creating uniform, equal-sized groups for each of the four categories. The groups are then de-indexed, meaning they return to their original position in the sequence. The third binning strategy follows an exponential approach, in which the width of each one of the four categories is determined by an exponential function. Each subsequent category is wider than the previous one, creating an increasing distribution of the data across categories. 

We began our analysis by assessing the robustness of the three binning strategies used to transform continuous emotion scores into discrete categories to be used as input into the CSSR algorithm. Since our goal was to model the complexity and uncertainty of communication dynamics, it is important that the binning process preserves the essential distributional characteristics of the data without introducing artifacts. We therefore evaluated all three binning strategies (quartile-based, ranked uniform, and exponential) using standard statistical diagnostics, including assessments of normality, heteroscedasticity, and multicollinearity in the resulting regression models. Among the three, the quartile-based binning strategy consistently outperformed the others, producing more stable model assumptions and better-aligned distributions across our sequences. As such, we adopted the quartile-based strategy for all subsequent analyses. The results reported below are based on this approach.

### 3.2. Dependent Variables

**Figure 1:**
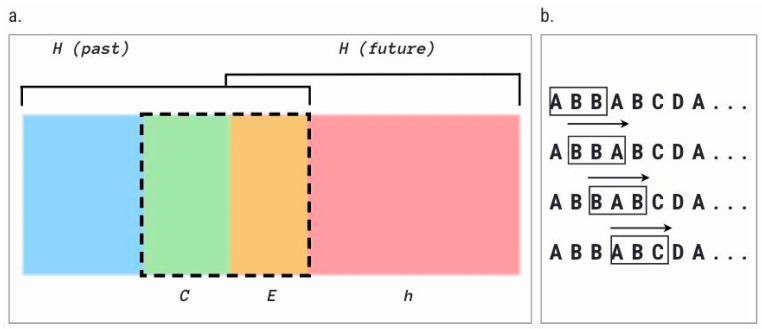
Diagram depicting the relationship between predictive information E, predictive complexity C, and remaining uncertainty. (**a**) The amount of information communicated from the past to the future through repeating patterns is quantified with *E*, the predictable information (the mutual information between the past and the future). The amount of information required to produce E is quantified by the complexity of the pattern, *C* (also known as the statistical complexity). The remaining uncertainty, *h*, is the entropy rate, which quantifies the amount of uncertainty of the future dynamic that cannot be predicted by identifying all possible patterns contained in the past. Graph adapted from [32,103]; (**b**) The illustration depicts the sliding-window approach we employed to measure the frequency statistics from character sequences. The observed frequency of these subsequences, which are essentially the fundamental components of the dynamic process, provide us with the statistics that outline the features of the underlying temporal structure, with a focus on how information is being preserved, created, and destroyed at each moment of the dynamic.

We calculated C and h for each of the 650 temporal sequences. To derive C, we used an empirically validated algorithm to compute the two measures from our data, namely, the Causal State Splitting Reconstruction (CSSR) algorithm [98], which reliably infers the statistical structure of a given dynamical process. We used Darmon’s validated and proven implementation and adopted those default settings [97,99]. It produces three outputs: predictive information *E*, predictive complexity *C,* and remaining uncertainty *h*, though in this study, we are mostly concerned with complexity and uncertainty. All variables are measured in bits, which gives the optimal or average minimum number of yes-or-no questions that an observer would have to ask to completely reconstruct a system. The below Venn diagram, based on Crutchfield et al. [32], portrays the relationship between these three complementary measures along temporal sequences of past and future (Figure 1a). 

Within this formalism, predictive information is the common information between past and future states; that is, the information preserved by the dynamical system over time. The predictive complexity (C) includes the information from the past that is necessary to predict the predictable component of the future (the information necessary for the ‘machinery’ to make predictions, hence, the ‘complexity’), and the remaining uncertainty (h) is the amount of information about the future that cannot be derived from the past behavior (possibly random, possibly predictable on the basis of information external to the model’s reach). To derive the statistical measures of character sequences in our data, we used sliding windows of three characters as depicted in Figure 1b and measured the occurrence of unique character subsequences. These measures are calculated with the Python 3 implementation of CSSR by Darmon [98].

#### 3.2.1. Predictive Complexity (C)

Predictive complexity, expressed with *C,* is also known as statistical complexity [97] and approximates Kolmogorov complexity [96]. It quantifies the minimum amount of information that can predict the amount of structure from the past that is useful to predict the future (or “predictable information”, *E* in Figure 1a). Essentially, it is measured as the number of bits needed to optimally predict the future of the process from its past [32]. For instance, *C* would be zero for an entirely random process. The higher the required number of bits to predict the future, the more complex the process is. Complexity is therefore defined as the information required to describe a process “between order and chaos” [96]. It corresponds to the creation or preservation of information or structure in the signal. The mathematical beauty of the measures is that it is shown that the predictive complexity is a sufficient statistic, with the minimal size representation of the structure, but with maximal predictability of what can be predicted about the process [97,104]. Hence, C is a practical approximation of the Kolmogorov complexity of a dynamic, in the sense that it measures the size of the smallest model with maximal predictive power for the time series [96]. Technically, it is measured as the entropy of the derived epsilon machine, i.e., the Shannon entropy over the causal states of the hidden Markov model that represents the dynamic C = H [Causal States].

#### 3.2.2. Remaining Uncertainty (h)

The remaining uncertainty, expressed with the traditional measure of the entropy rate *h,* is a fundamental and the oldest information-theoretic measure of dynamical systems [29,31]. It quantifies the amount of uncertainty per symbol about the future that remains in a process, given all the information the previous state of the process can tell us about the future. It is essentially the rate of conditional entropy *H* calculated per symbol, h = H[X|X_0_], measuring the uncertainty involved in predicting the next symbol based on the previous one [28]. The resulting entropy rate measures the uncertainty of the next turn conditioned on the previous turns [105]. In short, the entropy rate “gives the source’s intrinsic randomness, discounting correlations that occur over any length scale” [96]. Naturally, the higher the entropy rate, or remaining uncertainty, the higher the probability of a prediction error, which makes the future of the process less predictable [101].

### 3.3. Independent Variables

The main independent variable in our study is botness, or “bot level”, the extent to which an account is estimated to be a bot. To assess the automation level involved with each account, we used a publicly available bot detection service frequently used in previous work, the Botometer API [67,106]. Using machine learning algorithms, Botometer extracts over 1000 predictive features that identify numerous suspicious behaviors by characterizing an account’s profile, social network, friends, temporal activity patterns, language and sentiments [70,106]. It then provides a classification score on a normalized scale, indicating the probability that a Twitter account is likely to be a bot. Scores closer to 1 indicate a higher probability of the account being a bot, while those closer to 0 suggest that the account is controlled by a human. We averaged the resulting scores for each tweet per sequence. The variable bot-level measures the percentage of *botness* within each of the 650 temporal sequences made up of 3000 consecutive tweets (M = 0.045, SD = 0.008, min = 0.024, max = 0.081).

The continuity of this measure captures not only uncertainty about the conclusion that an account is a bot account, but also the fact that accounts vary in their botness, as in the case of accounts that tweet a mix of human and automated content. This continuous scale allows us to account for the presence of hybrid or semi-automated accounts that exhibit both human and bot-like behaviors, a common occurrence in political discourse. By using an averaged score across tweet sequences, we incorporate both subtle and overt forms of automation into our analysis, rather than relying on a binary classification threshold.

We also control for several potential confounding variables to account for the characteristics of tweets. One such confounding factor that could affect the complexity and uncertainty of a communication dynamic is the number of words used. This measure could contribute to complexity, for longer tweets with more words could contain multiple clauses with complex sentence structures, potentially increasing the level of information contained. The variable “word count” is measured by calculating the number of words used in each tweet and averaging the total count per each temporal sequence (M = 21.219, SD = 0.456, min = 19.879, max = 24.18). 

Another factor we consider is the expression complexity per word. Longer and more complicated words used in tweets can indicate a more complex sentence structure, while shorter or simpler words can drastically reduce the amount of information conveyed. The variable “word complexity” is created by dividing the number of characters in each tweet by the number of words per tweet, averaged per sequence (M = 6.497 SD = 0.127, min = 6.318, max = 6.778). 

Finally, we control for time variation between tweets, for a high time variance could make it more difficult to predict how future states will be related to the past, which can affect the overall uncertainty and predictability. We therefore measure the variable “time-variance” to control for the variation of time between the first and last tweets across each temporal sequence (M = 14.4, SD = 117.5, min = 0.477, max = 1338.7).

## 4. Results

**Figure 2:**
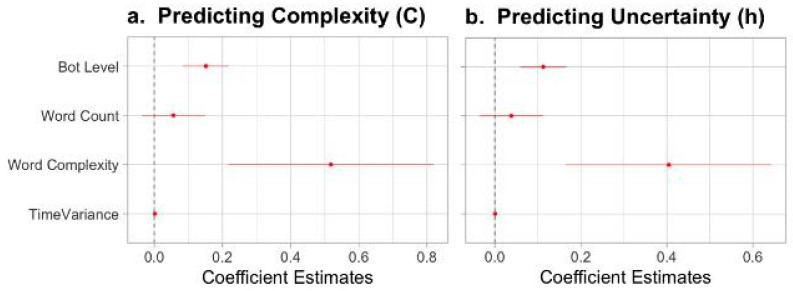
Standardized regression coefficients for (**a**) predicting complexity and (**b**) remaining uncertainty bound with 95% confidence intervals.

We first assessed the relationship between bot presence and the predictive complexity, reflecting how much information from the past is required to foresee the future state. Overall, the regression model is significant (F(5, 641) = 38.73, *p* < 0.001, $R^{2}$= 0.232, Figure 2a). Our results suggest a significantly positive relationship between the presence of bots and predictive complexity (β=0.151, p<0.001, Figure 2a). Notably, higher levels of bot presence correspond with higher levels of complexity. Put simply, online political communication sequences with higher bot levels require more past information to predict future behavior. The presence of bots creates more complex patterns.

Additionally, we found that both the complexity of the language used in tweets, or word complexity, (β=0.517, p<0.001) and the time variance (β=0.003, p<0.001) also positively and significantly contribute to the predictive complexity of the ongoing discussion, though the contribution of time variance is comparatively much smaller. Moreover, the results show a positive relationship between word count and predictive complexity, but this relationship lacked significance (β=0.055, p=0.23).

Turning to the remaining uncertainty involved in the online political discourse, our second model is also significant (F(5, 641) = 35.08, *p* < 0.001, $R^{2}$= 0.214, Figure 2b). Notably, higher bot levels corresponded with increased levels of uncertainty (β=0.113, p<0.001). Controlling for all the other predictors we included in the model, higher bot levels significantly correlate with higher remaining uncertainty: the higher the bot levels in sequences of tweets, the larger the component of future behavior that cannot be predicted from past states. As for the control variables, we found a positive relationship between uncertainty and word complexity (β=0.404, p<0.001) and a small effect from time variance (β=0.001, p<0.001). However, the positive relationship between word count and uncertainty lacked significance (β=0.04, p=0.03).

## 5. Discussion

This study sheds light on the structural changes in online political discussion dynamics attributable to bot activity by analyzing the relationship between bot presence, predictive complexity, and remaining uncertainty. Our results illuminate the role of political bots in macro-level dynamics of online political discourse, demonstrating that increased bot activity is associated with higher levels of structural complexity and uncertainty.

By applying Claude Shannon’s information theory [28] within a dynamical systems framework, we offer a novel lens through which to view online political communication. Contrary to their intended function of simplifying communication through automation and repetition [34,35,88], politically motivated bots’ presence is associated with increased complexity and uncertainty within online discourse. Our results suggest that higher bot activity is associated with a larger component of future behavior that cannot be predicted from the past states, indicating more uncertainty in the process.

It may seem counterintuitive that predictive complexity (C) and remaining uncertainty (h) both increase with bot presence: since C measures the structure of a process that allows for predictability, why would it also increase uncertainty? The solution consists in the recognition that uncertainty is not a finite quantity (like ‘a given box’) that can be saturated by predictability (like ‘filling up that given box’), but there is an infinite amount of both possible uncertainty and predictable structure in reality (like a ‘box that grows with its own structure, as does the unfilled room inside it’). This co-occurrence is characteristic of many complex adaptive systems. In purely random systems, h is high but C is low, while in fully deterministic systems, both values can be low. However, semi-structured systems, such as political discourse shaped by both human behavior and automated interference, often exhibit both high structure and high unpredictability. In our case, bots may introduce organized temporal or emotional patterns (e.g., coordinated amplification) that increase C, while simultaneously destabilizing semantic flow or introducing unanticipated variation, which increases h.

Technically, C measures the entropy of the causal states of the minimal-size-maximally-predictive hidden Markov model of the dynamic (its so-called epsilon machine) [96,97]. That quantifies the number of causal states and the inequality of their distribution. C increases if there are more states and if their distribution is more equally distributed. The entropy rate, h, is captured by the transition probabilities between the different causal states. It quantifies the uncertainty from transitioning between the states. If there are more states, there are often also more possibilities to transition between them. Hence, often (but not necessarily), both C and h increase in complex systems. Our chosen framework illustrates that C and h reflect distinct and complementary aspects of information flow in complex communicative environments.

In practical terms, the usual cues and patterns that individuals rely on to interpret messages and intentions online could become obscured. The presence of bots introduces noise and unpredictability, which could affect the ability of users to engage in reasoned debate or to follow the progression of events online. This may not only affect the immediate understanding of specific conversations or the perceptions of certain critical political events but could also erode trust in the digital communication environment as a whole, as users increasingly become uncertain about which interactions are genuine and which are manipulated to control public opinion [13,84,86]. 

Higher bot activity also requires more past information to predict the same amount of future behavior, indicating a greater degree of complexity in the process. This complexity is not necessarily a challenge in terms of the volume or density of information, but rather it shows a substantive change in the nature of the discourse itself. This additional layer of complexity could make it more difficult for both individuals and algorithms (such as those powering recommendation systems) to filter and understand the essential elements of the ongoing conversations. Earlier work shows that bots can substantially shape discussions by influencing search engines and recommender systems [81], and their impact on communication dynamics may exacerbate these effects. Moreover, bots’ potential ability to skew the semantic direction of the discourse may distort the public’s perception of consensus or controversy on critical issues, further polarizing audiences. The outcome is a further distorted representation of public opinion, where artificially amplified themes and emotions gain undue prominence, disrupting discussions and potentially misleading observers about the true nature of public sentiment around controversial topics. 

Our analysis also reveals that the complexity of the language used in tweets (word complexity) and the intervals between posts (time variance) also significantly influence both the complexity and uncertainty of online political discussions, though the influence of time variance is rather weak. Conversely, the volume of words used (simple word count) does not show significance. These results align with and support previous research emphasizing the value of quality engagement in sustaining the vitality and success of online communities and digital platforms, over frequency or quantity of engagement [107,108,109,110].

The present study makes several important contributions. Our study’s focus on the macro-level dynamics of an online political discussion provides an important expansion of the current work on social bots. Previous research has largely focused on the prevalence, reach, detection, and influence of social bots, such as their role in spreading misinformation [8,15,17], interfering in political campaigns [7,8,10], and amplifying polarizing viewpoints [58,64]. While these studies provide crucial insights, they often overlook the broader structural implications of these automated entities on the digital ecosystem. By analyzing how bots contribute to the structural complexity and uncertainty of online political discourse, our work offers a holistic view of their influence, underscoring how bots fundamentally alter the dynamics of online political communication. 

Our study also contributes to the growing literature on information theory in understanding communication processes. By extending the dynamical systems framework to political bots and using information-theoretic measures to quantify the complexity and uncertainty of online political communication, we expand on the previous work and provide a novel approach to studying the impact of political bots. This approach could be further extended to other domains, such as crisis communication and health communication, and could provide insights into the macro-level dynamics of online communication that previously went unexplored.

Furthermore, this study applies the Causal State Splitting Reconstruction (CSSR) algorithm [111] to analyze online political communication. This algorithm has previously been used in other fields to analyze dynamical systems, and our study demonstrates its potential for use in the analysis of online communication on social media platforms.

While our results demonstrate strong associations between bot presence and both increased complexity and uncertainty in political discourse, the causal direction of this relationship remains open to interpretation. Bots may actively shape communicative dynamics by introducing structural disruptions, but it is also possible that already fragmented or emotionally charged conversations attract greater bot activity. Moreover, external events, such as debate timing or real-world controversies, could simultaneously drive both complexity in user discourse and increased bot deployment. Although our sampling strategy and theoretical rationale support the interpretation that bots influence discourse dynamics, future research using longitudinal, experimental, or time-resolved network methods is needed to disentangle these causal pathways with greater precision.

## 6. Conclusions

This study provides evidence for the macro-level impact of political bots using a dynamical systems framework. By applying information-theoretic measures to sequences of emotionally coded tweets, we show that political bots are associated with higher levels of both structural complexity and unpredictability in online political discourse. These results highlight the importance of studying communication processes as complex and dynamic systems and the need for further research into the macro-level influence of bots.

It is important to note that we cannot establish a causal relationship among the variables we considered in this work, and there is a chance that a different variable, beyond those we control for in this study, plays a causal role. Furthermore, our study only provides a snapshot of an online political discussion, and the Twitter API we used in data collection only provides 1% of the total traffic on Twitter. This limited sample may not fully capture the dynamics of online political communication, and findings may not generalize across different platforms, events, or contexts. Each platform’s unique dynamics and algorithms can influence bot and user behavior differently. Further research is needed to understand the broader effects of bot activities on various social and political discussions.

In addition, while we rely on the well-established Botometer tool to estimate bot presence, we acknowledge that the reliability of bot detection can vary over time. As social media platforms evolve and new forms of automation emerge, the features used by detection algorithms may become outdated or less effective. Consequently, our botness scores should be interpreted as probabilistic indicators of automation, not definitive classifications. The use of continuous bot scores allows us to capture both overt and subtle forms of automated behavior across sequences, making our findings robust to minor classification fluctuations.

Despite these limitations, the results point to an urgent need to better understand, and design for, the structural consequences of bots on online political conversations. As bots become increasingly prevalent and sophisticated, their influence is likely to extend far beyond individual interactions, shaping the very fabric of collective discourse. This is especially true in an age of generative AI agents, which exhibit far greater autonomy now than ever before. By adopting a complex systems perspective on bots and online political communication, our presented framework showcases how this can be done. Dynamical systems approaches can provide a nuanced and comprehensive understanding of the interplay between technology and society in the digital age.

## Data Availability

The raw data supporting the conclusions of this article will be made available by the authors on request.
